# Modified Arrhenius Equation in Materials Science, Chemistry and Biology

**DOI:** 10.3390/molecules26237162

**Published:** 2021-11-26

**Authors:** Jan Kohout

**Affiliations:** Department of Mathematics and Physics, Military Technology Faculty, University of Defence, Kounicova 65, CZ-662 10 Brno, Czech Republic; jan.kohout@unob.cz; Tel.: +420-973-443-283

**Keywords:** Arrhenius equation, curved Arrhenius plot, curved Kissinger plot, austenitization kinetics, crystallization kinetics, food spoilage kinetics, growth rate of bacterial cultures, plant leaf respiration rate, temperature dependence of ant creeping, temperature dependence of heartbeat rate

## Abstract

The Arrhenius plot (logarithmic plot vs. inverse temperature) is represented by a straight line if the Arrhenius equation holds. A curved Arrhenius plot (mostly concave) is usually described phenomenologically, often using polynomials of *T* or 1/*T*. Many modifications of the Arrhenius equation based on different models have also been published, which fit the experimental data better or worse. This paper proposes two solutions for the concave-curved Arrhenius plot. The first is based on consecutive A→B→C reaction with rate constants *k*_1_ ≪ *k*_2_ at higher temperatures and *k*_1_ ≫ *k*_2_ (or at least *k*_1_ > *k*_2_) at lower temperatures. The second is based on the substitution of the temperature *T* the by temperature difference *T* − *T*_0_ in the Arrhenius equation, where *T*_0_ is the maximum temperature at which the Arrheniusprocess under study does not yet occur.

## 1. Introduction

The Arrhenius equation was published in 1889 [1]. It describes very accurately the temperature dependence of the kinetics of chemical reactions of simple chemicals (cane sugar was studied in the cited paper). According to the Arrhenius equation, the reaction rates at very low temperatures are very small but non-zero. The Arrhenius equation is also used in materials science and biology, for example in describing the kinetics of austenitization [2], the respiration rate of plant leaves [3], or the heartbeat rate of terrapins [4]. In these cases, kinetics at low temperatures are meaningless: austenitization can only take place at temperatures above Ac_1_ and many living organisms die or at least their bodily processes stop at body temperatures below freezing. This seems to be one of the main reasons for the invalidity of the Arrhenius equation, which is manifested by the curved Arrhenius plot.

The Arrhenius equation for rate constant *k* is

(1) $$k=k_{\infty }\exp\left(-\frac{E}{RT}\right)$$

where pre-exponential factor *k*_∞_ is formally rate constant for infinite temperature, *E* is the activation energy, *R* is the universal gas constant and *T* is the absolute temperature. After logarithmization

(2) $$\ln k=-\frac{E}{R}\cdot \frac{1}{T}+\ln k_{\infty }$$

a linear dependence of ln*k* on 1/*T* with slope −*E*/*R* is obtained, which is represented graphically is called the Arrhenius plot.

Differential scanning calorimetry (DSC) is used in many phase transformations studies when samples are heated (or cooled) over a wide range of heating (or cooling) rates. Maximum heat flow for different rates *β* is obtained at different “peak” temperatures *T*_p_. The dependence of *β* on *T*_p_ is described by the Kissinger equation (see e.g., [5])

(3) $$\ln\frac{\beta }{T_{p}^{2}}=-\frac{E}{R}\cdot \frac{1}{T_{p}}+C$$

where *C* is a constant coefficient. From a formal point of view, the Kissinger and the Arrhenius equations are the same types of dependence, so the results of the DSC examination, which are readily available, can be used for further considerations.

The Kissinger equation, published in 1957 [6], received many thousands of citations and Kissinger became probably the most important figure in the field of differential thermal analysis (DTA) [7]. Šesták et al. [8,9] accounted for the thermal inertia component of the heat flow omitted by Kissinger, leading to a more accurate determination of the activation energy and to distinguish the temperature of the extreme deviation of the DTA from the temperature at which the reaction rate is maximum. For the purposes of the presented paper, Equation (3) is sufficient. Temperatures corresponding to the maximum reaction rates would be useful, but unfortunately, the authors of the papers whose data are used for regression calculations presented here have not mentioned their values.

**Figure 1:**
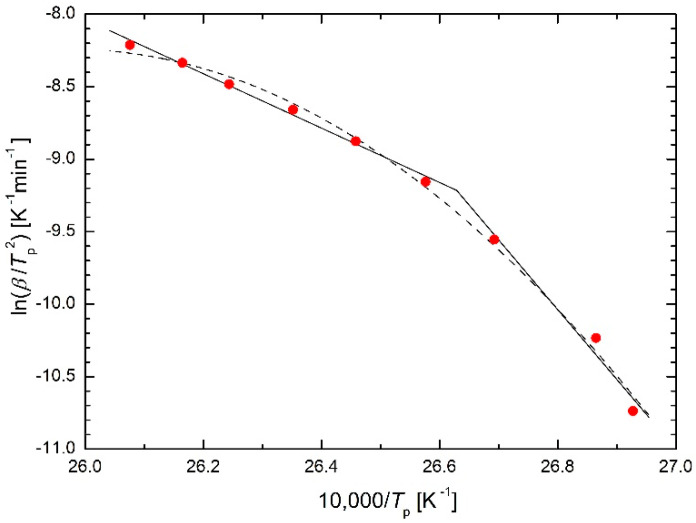
“Double” Kissinger plot for Ni-Mn-In-Mg shape memory alloy (see [5], Figure 7, solid line) and Kissinger plot based on the analogy of the Equation (5) (dashed line).

Zhou et al. [5] studied the kinetics of martensitic transformations in Ni-Mn-In-Mg shape memory alloys and obtained a curved Kissinger (Arrhenius) plot. They decided to fit it using two straight lines, see Figure 1 (solid line).

This is the simplest approach, giving two very different activation energy values, 156 kJ/mol and 402 kJ/mol. However, it raises more questions than answers:

- In terms of materials science: why is the activation energy for heating rates higher than 9.2 K/min 2.5 times lower than for heating rates lower than 9.2 K/min? What is the reason for this step change?
- In terms of regression: is it correct to replace an almost smooth arc with two straight lines? If, in a study [5], the heating at heating rates of, e.g., 40 K/min and 35 K/min is not performed and heating at, e.g., 2 K/min and 1 K/min is added, then very likely the straight lines in the Kissinger plot will change their positions substantially, i.e., not only the two values of activation energy will change substantially, but also the value of heating rate separating these straight lines. What is the usefulness of the results obtained in this way?

Deviation from the linear shape of the Arrhenius plot (from “Arrhenius behaviour”) is often referred to as the “non-Arrhenius behaviour” [10] and is distinguished into the “super-Arrhenius behaviour” (concave curve) and “sub-Arrhenius behaviour” (convex curve), e.g., [10,11]. Many authors distinguish only between “concave Arrhenius plot” and “convex Arrhenius plot”, but some of them interchange the terms “concave” and “convex”, e.g., [12]. Many models have been published, especially recently, but almost without exception they are extremely complicated, designed for a very specific scientific problem, without any generality. The aim of this paper is to find a model or relation with similar generality to the original Arrhenius equation.

One of the classical approaches to describing curved Arrhenius plots consisted in a phenomenological description of their shape, mostly using several initial terms of convergent mathematical series in the Arrhenius equation. These were sometimes called “extended Arrhenius equations”, e.g., in [13]. Dozens of papers with extended Arrhenius equations were published especially in the 1970s and 1980s. For example, a logarithmic equation with coefficients *A* to *E* (or more) was published by Kanerva et al. [14]:

(4) $$\ln k=A+B/T+C\ln T+DT+ET^{2}+\ldots$$

where the extension consisting in the polynomial of *T* is supplemented by a *C*ln*T* term leading to a multiplicand *T^C^* in the original equation. Yang et al. [15] also published a logarithmic equation

(5) $$\ln k=\ln A+\frac{B}{RT}+\frac{C}{{\left(RT\right)}^{2}}$$

where the extension consists of an additional term of the polynomial of 1/*T*. All of these equations containing three or more parameters usually give a very good fit, but their regression parameters have no physical meaning, i.e., their meaningful interpretation is virtually impossible. An example application of Equation (5) (in fact, its analogy) is given in Figure 1 (dashed line).

Some authors have tried to solve the curvature of the Kissinger plot theoretically. Elder [16,17] suggested adding a term *T^m^* in the Kissinger equation to obtain the so-called generalized Kissinger equation:

(6) $$\frac{d\alpha }{dt}=AT^{m}\exp\left(-\frac{E}{RT}\right)f(\alpha )$$

where *α* is the dimensionless degree of reaction or transformation and *f*(*α*) is the suitable function that can be described by a suitable dependence on *T*, *R*, and *E*. Parameter *m* can take the following values [16,17]:

- *m* = 0 leads to the standard Kissinger equation (the Arrhenius assumption is followed [16])
- *m* = 0.5 is predicted by collision theory in the homogeneous gas phase and
- *m* = 1 is predicted by transition state theory.

The author of this paper attempted to use Equation (6) as a regression function in many cases of curved Arrhenius or Kissinger plots, but values of *m* = 0.5 or *m* = 1 did not noticeably change the linearity of these plots. Some curvature appeared for values of *m* in the tens, hundreds, or even thousands, but such values of *m* have no physical meaning. Formally, Equation (6) corresponds to the Equation (4) in which the first three terms are considered.

**Figure 2:**
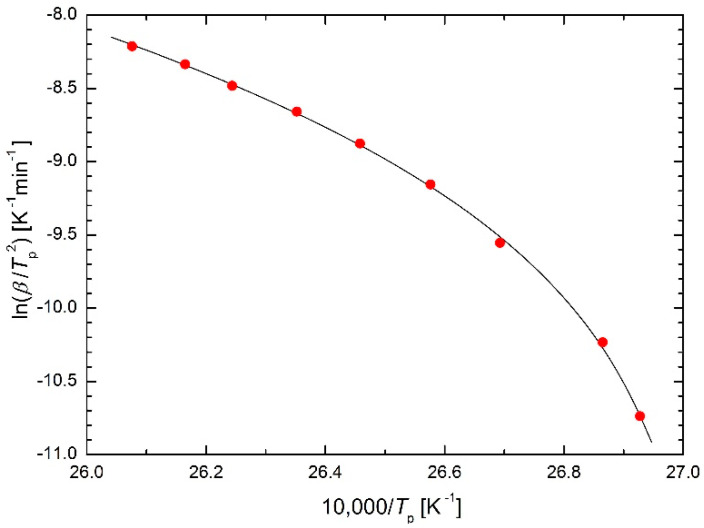
Kissinger plot for Ni-Mn-In-Mg shape memory alloy [5] using Equation (7) as regression function.

Norwisz [18] and, apparently independently, Dutta and Ryan [19] modified the Kissinger equation into the form:

(7) $$\frac{d\alpha }{dt}=A\exp\left(-\frac{E}{RT}\right)\left[1+\frac{E}{RT}\left(1-\frac{T_{0}}{T}\right)\right]f(\alpha )$$

for DSC linear heating with time *t*:

(8) $$T=T_{0}+\beta t$$

where the initial temperature of heating *T*_0_ is taken into account. This modification is indeed able to curve the Arrhenius plot, but it contains one logical weakness: if the heating starts sufficiently low below the temperatures at which the reaction or transformation under study takes place, the initial heating temperature *T*_0_ may have hardly any noticeably effect on the kinetics of the process, whereas it plays a crucial role in Equation (7). To clarify the role of *T*_0_ in Equation (7), the experimental data from [5] were fitted using Equation (7) as a regression function where *T*_0_ is the calculated regression parameter. The successful regression is shown in Figure 2 whereas the calculated value of parameter *T*_0_ is quite surprising: *T*_0_ = 396 K. Figure 6 in [5] shows that this temperature value *T*_0_ cannot be the initial temperature of heating, but it is the temperature at which the heat flux in DSC returns to its constant value after reaching the maximum value, namely for the highest heating rate. In other words, this temperature characterizes the end of the transformation process. From this point of view, Equation (7) can hardly be considered a useful result of theoretical derivation, rather it is a phenomenological equation of the type:

(9) $$\frac{d\alpha }{dt}=A\exp\left(-\frac{E}{RT}\right)\cdot \left(1+\frac{E}{RT}-\frac{B}{T^{2}}\right)f(\alpha )$$

or:

(10) $$\ln k=\ln A-\frac{E}{RT}+\ln\left(1+\frac{E}{RT}-\frac{B}{T^{2}}\right)$$

representing only another variant of phenomenological Equations (4) and (5).

A possible explanation for the deviation from the Arrhenius equation is that the reaction $A\overset{k}{\to }C$ does not proceed through a single-step rate-determining mechanism, but proceeds through an intermediate B, e.g., as follows [12]:

(11) $$A\overset{k_{1}}{\underset{k_{-1}}{⇄}}B\overset{k_{2}}{\to }C$$

Then for the rate constant *k* of the reaction as a whole, one can write [12,20]:

(12) $$k=\frac{k_{1}k_{2}}{k_{-1}+k_{2}}$$

This equation can be simplified:

(13) $$\begin{array}{c} k=k_{1}\;\mathrm{for}\;k_{2}\gg k_{-1}\\ k=\frac{k_{1}k_{2}}{k_{-1}}\;\mathrm{for}\;k_{2}\ll k_{-1} \end{array}$$

The reaction scheme (11) is probably the simplest published scheme used for this reason; a number of more complex schemes can be found in some papers.

Based on Equations (11)–(13), the author of this paper proposed a simpler scheme:

(14) $$A\overset{k_{1}}{\to }B\overset{k_{2}}{\to }C$$

where:

(15) $$\begin{array}{c} k=k_{1}\;\mathrm{for}\;k_{2}\gg k_{1}\\ k=k_{2}\;\mathrm{for}\;k_{2}\ll k_{1} \end{array}$$

Then the analogy of the Equation (12) can be reconstructed retrospectively:

(16) $$k=\frac{k_{1}k_{2}}{k_{1}+k_{2}}$$

where the Arrhenius equation can be used for *k*_1_ and *k*_2_. The resulting regression function then contains the four regression parameters *k*_∞1_, *k*_∞2_, *E*_1_, and *E*_2_. However, for better stability of regression calculations, it is better to write the relations for rate constants *k*_1_ and *k*_2_ in the form:

(17) $$k_{i}=k_{12}\exp\left[-\frac{E_{i}}{R}\left(\frac{1}{T}-\frac{1}{T_{12}}\right)\right],\;i=1\;\mathrm{and}\;2$$

where *T*_12_ is the temperature at which *k*_1_ = *k*_2_ = *k*_12_. For a concave-curved Arrhenius plot (super-Arrhenius behavior) *E*_1_ < *E*_2_, then *k*_1_ < *k*_2_ for *T* < *T*_12_ and *k*_1_ > *k*_2_ for *T* > *T*_12_. Then the resulting regression function contains the four regression parameters *k*_12_, *T*_12_, *E*_1_, and *E*_2_.

**Figure 3:**
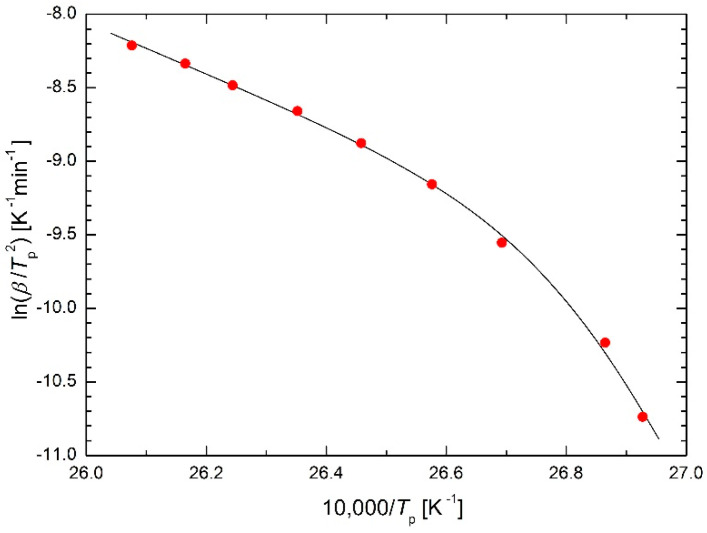
Kissinger plot for Ni-Mn-In-Mg shape memory alloy [5] using Equations (16) and (17) as regression functions.

Again, the experimental data from [5] were fitted to validate the presented approach, see Figure 3. As it can be seen, the fitting is successful in this case as well, similar to Figure 2. The regression calculations give a value of temperature *T*_12_ = 372 K, which characterizes the position of the curve bending. The calculated activation energies of 143 kJ/mol and 799 kJ/mol can be compared with the values of 156 kJ/mol and 402 kJ/mol [5] obtained by fitting with two straight lines (see also Figure 1): while the *E*_1_ values are comparable, the *E*_2_ values are in a 2:1 ratio. The sensitivity to adding or removing experimental points at the edges of the test temperature range was also modeled. While the fit with two straight lines is extremely sensitive to such changes, the sensitivity of the fit using regression function (16) is substantially lower, but far from negligible.

Formally, the same procedure can be used for a convexly curved Arrhenius plot (sub-Arrhenius behavior), only the regression function (16) must be replaced by *k* = *k*_1_ + *k*_2_.

## 2. Derivation of the Modified Arrhenius Equation

The linearization plot is used not only in the case of the Arrhenius equation. The cumulative distribution function (cdf) of the Weibull distribution used in survival analysis, reliability engineering, failure analysis, etc., is as follows:

(18) $$F(x;a,b)=1-\exp\left[-{\left(\frac{x}{a}\right)}^{b}\right]$$

where *a* is the scale parameter and *b* is the shape parameter. This cdf can be linearized:

(19) ln{−ln[1−F(x)]}=blnx−blna

that is, the term on the left side of this equation is a linear function of ln *x*. The plot with axes:

(20) $$\ln x\;\mathrm{versus}\;\ln\left\{-\ln\left[1-\hat{F}(x)\right]\right\}$$

where $\hat{F}(x)$ is empirical cdf, is called the Weibull plot. If in a concave-curved Weibull plot, its left-end points to the ln*x*_0_ on the *x*-axis, it is an unmistakable indication that the three-parameter Weibull distribution:

(21) $$F(x;a,b,x_{0})=1-\exp\left[-{\left(\frac{x-x_{0}}{a}\right)}^{b}\right]$$

should be used, i.e., formally *x* is replaced by *x* − *x*_0_. By analogy, if in a concave-curved Arrhenius plot its right end tends towards the 1/*T*_0_ on 1/*T*-axis, the replacement of the temperature *T* by the temperature difference *T* − *T*_0_ should be considered in the Arrhenius equation:

(22) $$k=k_{\infty }\exp\left[-\frac{E}{R(T-T_{0})}\right]$$

For a three-parameter Weibull distribution, the relation *x* > *x*_0_ must be held. According to Equation (22), *k* = 0 for *T*→*T*_0_ but for many reasons it should be zero even for *T* < *T*_0_. Then Equation (22) must be replaced by the more accurate equation:

(23) $$\begin{array}{ccc} k=k_{\infty }\exp\left[-\frac{E}{R(T-T_{0})}\right] & & \mathrm{for}\;T>T_{0}\\ k=0 & & \mathrm{for}\;T\leq T_{0} \end{array}$$

**Figure 4:**
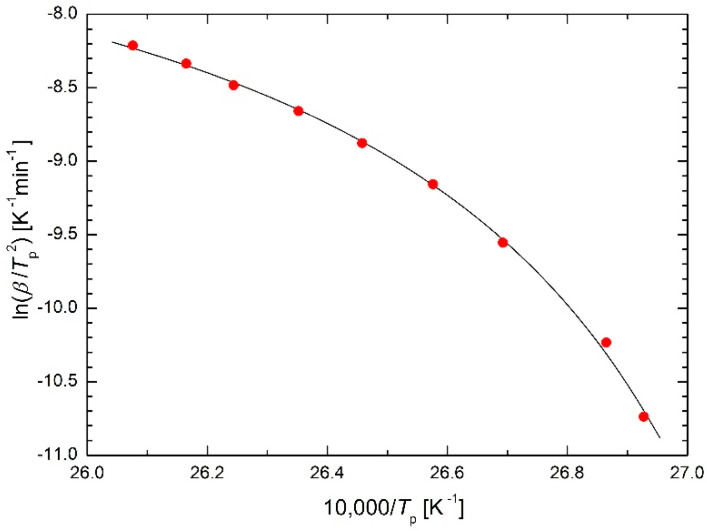
Kissinger plot for Ni-Mn-In-Mg shape memory alloy [5] using the modified Arrhenius Equation (23) as regression function.

Before discussing the newly derived regression function (23), let it be used for fitting the experimental data from [5], see Figure 4. It can be seen that the regression is again very successful. The regression calculations give *T*_0_ = 363 K. Figure 6 in [5] shows that at this temperature the heat flux in DSC starts to increase from its constant values at all heating rates. This means that this temperature is the initial temperature of the described transformation, in this case austenitization. Thus, only one parameter with a well-defined physical meaning has been added to the original Arrhenius equation (in the previous case they were two parameters), which causes the required curvature of the Arrhenius plot. This achieved the desired goal of this paper. It now remains to verify the suitability of the regression function (23) in the widest possible range of scientific research. Therefore, some specific cases will be presented.

Formally, for convexly curved Arrhenius plot (sub-Arrhenius behavior) the regression function:

(24) $$k=k_{\infty }\exp\left[-\frac{E}{R(T+T_{0})}\right]$$

can be applied (some of the first attempts look very promising). However, the physical interpretation of temperature *T*_0_ is likely to be a very difficult problem in this case.

## 3. Verification of the Suitability of the Modified Arrhenius Equation

### 3.1. Materials Science

The austenitization kinetics of SA 508 Gr.3 steel was studied by Luo et al. [2] using an isoconversional method. They described the dependence of the phase transformation rate on temperature using the Arrhenius equation (see Figure 5 in [2]):

(25) $$\frac{d\alpha }{dt}=A\exp\left(-\frac{E}{RT}\right)f(\alpha )$$

where *α* is the extent of conversion. The authors replaced the product *Af* (*α*) by a new pre-exponential factor *B*(*α*) and took into account the dependence of effective activation energy on the extent of conversion *E*(*α*):

(26) $$\frac{d\alpha }{dt}=B(\alpha )\exp\left[-\frac{E(\alpha )}{RT}\right]$$

**Figure 5:**
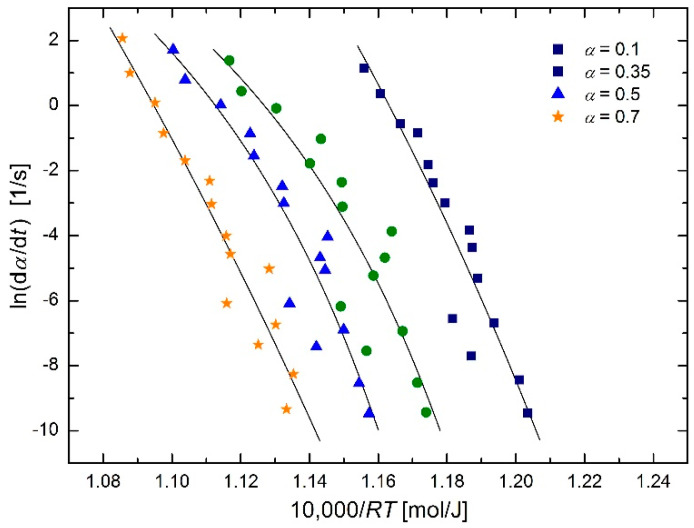
Arrhenius plot for austenitization of SA 508 Gr.3 steel using isoconversional method [2] based on the modified Arrhenius equation in the form of Equation (27).

Results of regression using the modified regression function:

(27) $$\frac{d\alpha }{dt}=B(\alpha )\exp\left[-\frac{E(\alpha )}{R(T-T_{0})}\right]$$

are shown in Figure 5; the temperature *T*_0_ values (means and standard deviations) for different extents of conversion *α* are given in Table 1.

**Figure 6:**
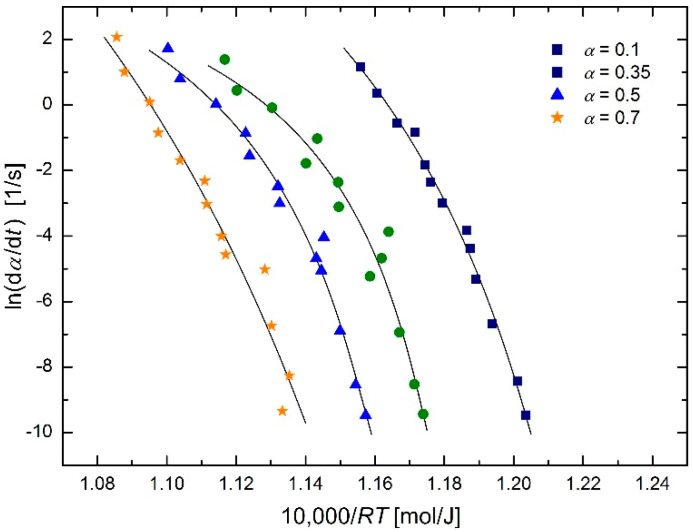
Arrhenius plot for austenitization of SA 508 Gr.3 steel using isoconversional method [2] (reduced data) based on the modified Arrhenius equation in the form of Equation (27).

Plots in Figure 5 show certain curvatures. However, a closer look at the figure reveals that every third and fifth lowest experimental point for all conversions appear to be outliers. Without performing any statistical test for outliers, these eight points (i.e., the points with *y*-values ≈ −6.3 and ≈ −7.5) were removed and the previous regression calculations were repeated. The results are shown in Figure 6 and Table 2.

**Figure 7:**
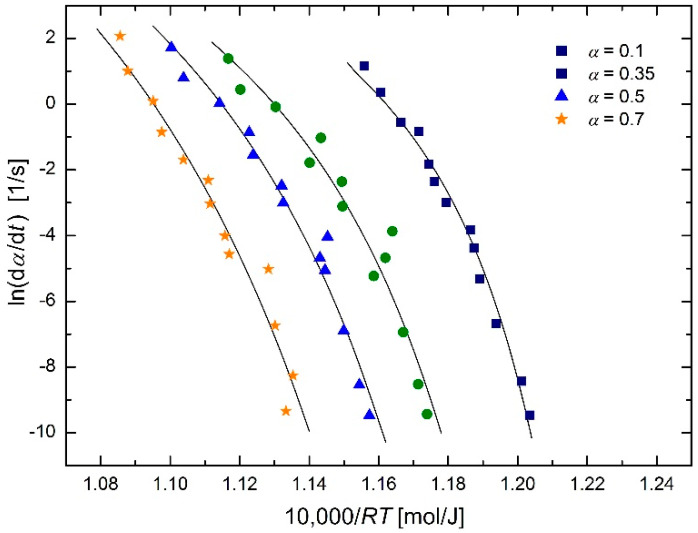
Arrhenius plot for austenitization of SA 508 Gr.3 steel using isoconversional method [2] (reduced data) based on the modified Arrhenius equation in the form of Equation (27), with common regression parameter *T*_0_ for all *α* values.

It can be seen that the *T*_0_ values in Table 2 are higher and closer together than the values in Table 1. Therefore, another regression of the reduced data set was performed, this time with a regression parameter *T*_0_ common to all four conversion values 0.1, 0.35, 0.5 and 0.7. This resulted in *T*_0_ = 966.8 K with an estimated standard deviation of 9.1 K, i.e., *T*_0_ = (967 ± 9) K = (694 ± 9) C. Graphical output of these calculations is shown in Figure 7.

Austenitization starts at temperature *Ac*_1_, which seems to be close to the obtained value of temperature *T*_0_. The temperatures *Ac*_1_ are not usually published, but they can be calculated from their chemical composition, which for SA 508 Gr.3 steel is given in Table 3.

For the calculation of the *Ac*_1_ temperature, the paper [21] was chosen, which is sufficiently new and takes into account as many elements from Table 3 as possible. It provides the equation:

(28) $$Ac_{1}=742-29\cdot \text{C}-14\cdot \text{Mn}+13\cdot \text{Si}+16\cdot \text{Cr}-17\cdot \text{Ni}-16\cdot \text{Mo}+45\cdot \text{V}+36\cdot \text{Cu}$$

where the symbols of chemical elements are to be replaced by their content in weight percent. Equation (28) for values gives *Ac*_1_ = 696.7 °C, with a recommended standard deviation of 9.2 °C [21], then the result can be written as *Ac*_1_ = (697 ± 9) °C = (970 ± 9) K. The agreement with the temperature *T*_0_ (see mean value in Table 2 and value *T*_0_ = (967 ± 9) K = (694 ± 9) °C obtained in common regression) is practically perfect. It can then be argued that the temperature *T*_0_ from the modified Arrhenius equation is directly *Ac*_1_ temperature of studied steel (logical judgment is supplemented by numerical verification).

**Figure 8:**
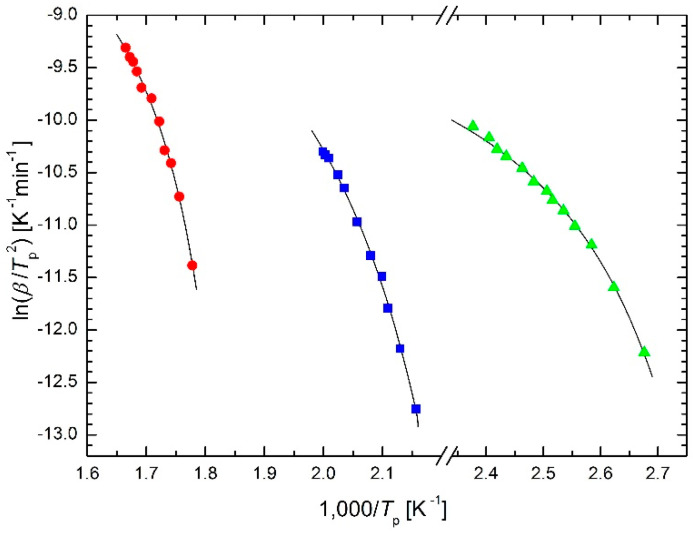
Kissinger plot for crystallization of Ga_7.5_Se_92.5_ chalcogenide glass [22] (triangles) and of Si_12.5_Te_87.5_ chalcogenide glass [23] (two separated crystallization peaks, squares and circles) based on the modified Arrhenius Equation (23).

DSC studies on the transformation kinetics of Ga_7.5_Se_92.5_ chalcogenide glass [22] (one crystallization peak) and of Si_12.5_Te_87.5_ chalcogenide glass [23] (two separated crystallization peaks) applying the isoconversional method were performed by El-Oyoun. The results are plotted in the Kissinger plots in Figure 8. The values of *T*_0_ are 339 K for Ga_7.5_Se_92.5_ chalcogenide glass, 413 K for the first crystallization peak and 522 K for the second crystallization peak, both for Si_12.5_Te_87.5_ chalcogenide glass. Comparing these temperatures with corresponding DSC curves [22,23], the *T*_0_ values characterize approximately the onset of heat flux for the lowest heating rates.

### 3.2. Food Storage

**Figure 9:**
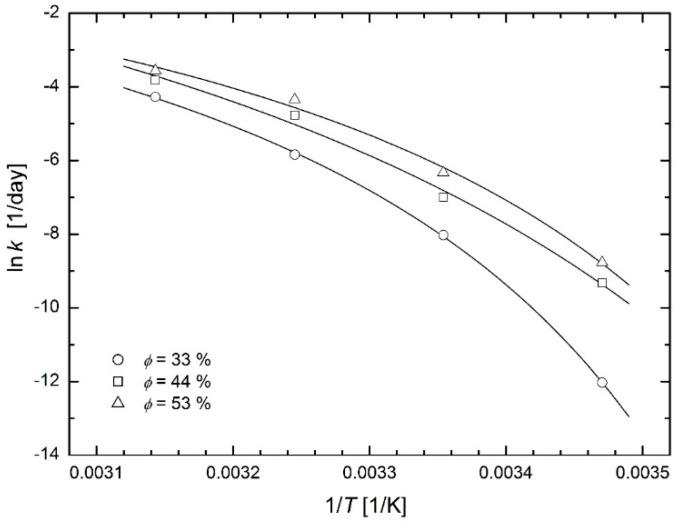
Modified Arrhenius plots for reaction rate constants of browning in freeze-dried onion at various relative humidity [24] (individual values of temperature *T*_0_ for each relative humidity).

**Figure 10:**
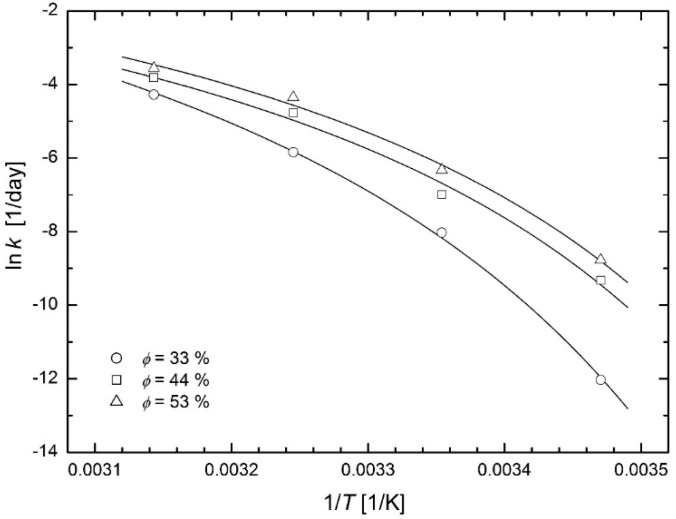
Modified Arrhenius plots for reaction rate constants of browning in freeze-dried onion at various relative humidity [24] (common value of temperature *T*_0_ = −18 °C for all values of relative humidity).

Sá and Sereno [24] studied the browning kinetics of onion and strawberry during the storage at temperatures 5, 15, 25, 35 and 45 °C and at relative humidity 33, 44 and 53 %. The extent of browning of the freeze-dried Portuguese red onion (*Allium cepa* L.) was determined by the measurement of the absorbance of the filtered extract in 1 cm quartz cells at wavelength of 420 nm using a UV/Vis Lambda 2 Spectrophotometer (Perkin Elmer, Connecticut, USA), see the official ADOGA method [25]. The authors could then determine the corrected reaction rate constants and plot them in the Arrhenius plot (see Figure 3 in [24]) with the Arrhenius straight lines and the curves corresponding to the Williams-Lander-Ferry model (WLF model) [26]. Figure 9 and Figure 10 in this paper show that the modified Arrhenius plot based on Equation (23) leads to a successful fit of the considered experimental data. Figure 9 shows fit with individual values of temperature *T*_0_ for each relative humidity: 261 K for 33%, 242 K for 44% and 255 K for 53%. Figure 10 shows the fits with the common value of temperature *T*_0_ = 256 K = −18 °C for all relative humidity values. Temperatures below −18 °C are used as a standard for long-term storage of food. The WLF model used by Sá and Sereno [24] to fit experimental data leads to the same fitting curves, as will be discussed in the *Discussion* chapter.

### 3.3. Botany

**Figure 11:**
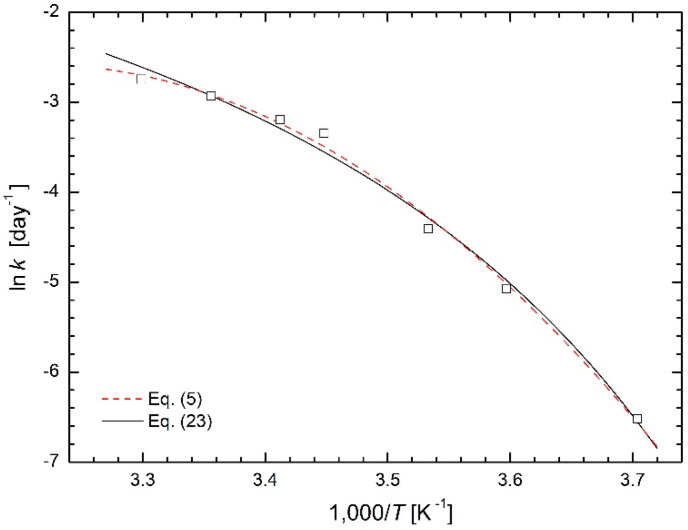
Modified Arrhenius plot for respiration rate of camellia leaves [3] based on Equation (23) is compared with the fit based on Equation (5).

Curved Arrhenius plots in the studies of biological processes (especially in the context of plants) were discussed by Wolfe and Bagnall [27] as early as 1980. In 44 references many models of various complexity explaining the curvature of the Arrhenius plots are given. Additionally, Nishiyama et al. [3] studied the temperature dependence of the respiration rate of camellia leaves (*Camellia japonica*) and obtained the curved Arrhenius plot. They compared the fitting using Equation (5) (see dashed line in Figure 11) with the split into two straight lines (it was experimental data of Nishiyama et al in [3] that were the basis for the paper of Yang et al. [15]). However, even in this case the modified Arrhenius Equation (23) gives a reasonably good fit, only slightly worse than Equation (5), see solid line in Figure 11. While Equation (5) is completely phenomenological and contains parameters without any physical meaning, the value of temperature *T*_0_ = 235 K = −38 °C obtained from the regression calculations using Equation (23) as regression function apparently represents the temperature below which respiratory processes no longer occur.

### 3.4. Microbiology

**Figure 12:**
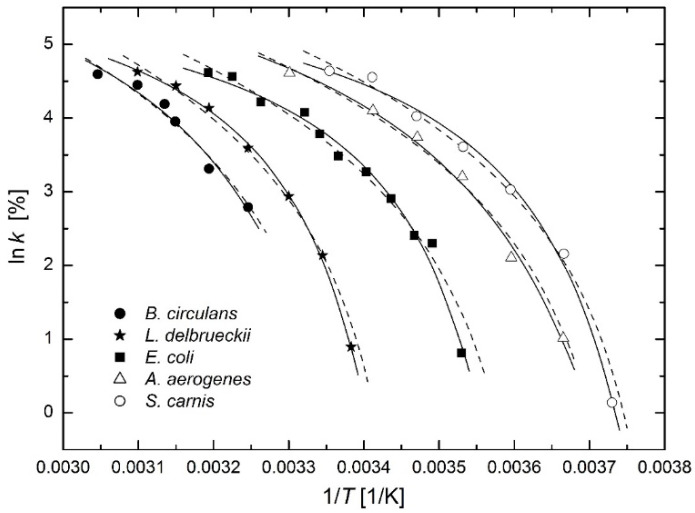
Arrhenius plots for the relative growth rates of bacterial cultures [28,29] based on the modified Arrhenius Equation (23) (solid lines) and on phenomenological regression function (29) (dashed lines). See Table 4 for full names of bacterial cultures.

Ratkowski et al. [28] drew the Arrhenius plots of the growth rates of bacterial cultures reported by Johnson et al. [29] (see p. 199). The growth rates of the studied bacterial cultures reach their maxima at certain temperatures. Johnson et al. [29] considered relative growth rates (maximum of growth rate = 100 %) and Ratkowski et al. [28] focused only on the temperature range where growth rates decrease with decreasing temperature (i.e., with increasing 1/*T*). They used fully phenomenological regression function:

(29) $$\sqrt{k}=b\cdot (T-T_{0})$$

proposed by Ohta and Hirahara [30]. In Figure 12 the Arrhenius plots of relative growth rates are drawn with fits based on the modified Arrhenius Equation (23) (solid lines) and on phenomenological regression function (29) (dashed lines). The modified Arrhenius Equation (23) leads to a better fit, especially for the highest growth rates. On the other hand, with regard to the number of regression parameters (only two) and the time of publication (1977), the function (29) represented a significant advance in the phenomenological description of growth of bacterial cultures at that time.

The values of temperature *T*_0_ obtained from regression calculations are shown in Table 4 for both regression functions (23) and (29). Additionally, the values published by Ratkowski et al. [28] are added for comparison. The negligible differences between the second to last column and the last column may be due to different regressions: *y* = ln*k* in this paper and *y* = $\sqrt{k}$ in paper [28]. Finally, it can be said that temperature *T*_0_ (in both Equations (23) and (29)) can be interpreted as the temperature below which the bacterial culture under consideration does not grow.

**Table 4:** Temperatures *T*_0_ for different bacterial cultures corresponding to the plots in Figure 12.

No	Bacterial Culture	*T*_0_ [K] (23)	*T*_0_ [°C] (23)	*T*_0_ [K] (29)	*T*_0_ [K] [28]
1	*Bacillus circulans*	283.4	10.3	296.0	296
2	*Lactobacillus delbrueckii*	283.6	10.5	290.9	290
3	*Escherichia coli*	271.3	−1.8	277.8	276
4	*Aerobacter aerogenes*	254.1	−19.0	267.4	267
5	*Sporotrichum carnis*	257.3	−15.9	264.7	264

### 3.5. Zoology

**Figure 13:**
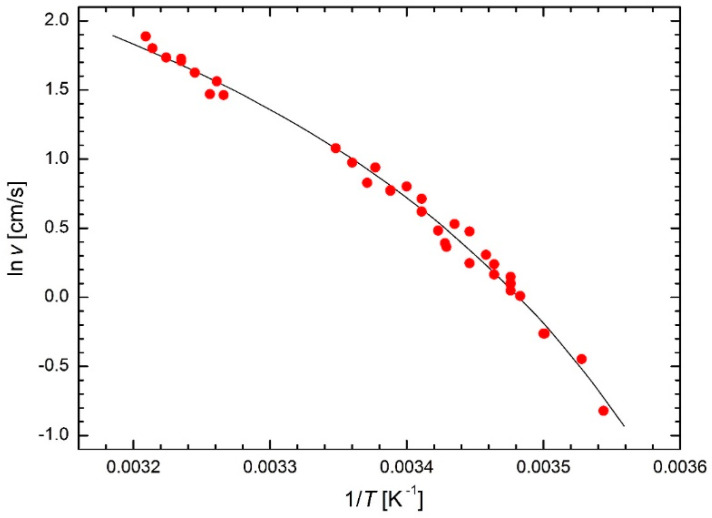
Modified Arrhenius plots for the speed of ant *Liometopum apiculatum* [31,32] based on Equation (23).

Laidler [4] summarized some unusual applications of the Arrhenius law in the animal kingdom, e.g., in entomology (chirping of tree crickets, creeping of ants or flashing of fireflies). In particular, the creeping of the ant *Liometopum apiculatum* (described by G. Mayr in 1870, widespread in southwestern United States and from northwestern to southeastern Mexico) using the Arrhenius law was studied by Crozier [31] who used the results of measurements of ant speed versus temperature made by Shapley [32]. The Arrhenius plot of ant speed values (excluding the two outliers specified by Shapley and the third outlier removed by Crozier) is shown in Figure 13. The fit based on the modified Arrhenius Equation (23) leads to temperature *T*_0_ = 252 K = −21 °C. It can be interpreted as the temperature at which the movement of the ants under study stops.

**Figure 14:**
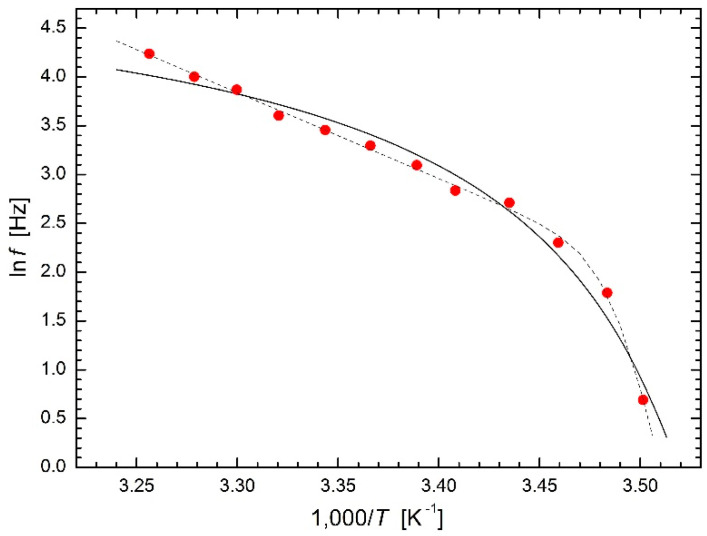
Modified Arrhenius plots for the rate of terrapin’s heartbeat [4,31] based on Equation (23) (solid line) compared with the fit using Equations (16) and (17) as regression function (dashed line).

Laidler [4] also presented the measurement of the frequency of terrapin’s heartbeat at different temperatures made by Martin [33]. The Arrhenius plot of the rate of terrapin’s heartbeat is shown in Figure 14. The experimental data are fitted excellently using Equations (16) and (17) as regression function, where temperature *T*_12_ = 285 K = 12 °C separates two nearly linear parts of the plot with different slopes, i.e., with different values of activation energy. A considerably worse fit is obtained using the modified Arrhenius Equation (23) but, on the other hand, regression parameter *T*_0_ = 277 K = 4 °C represents the limit temperature below which terrapin’s heartbeat appears to stop.

## 4. Discussion

The effort to cope with the curvature of the Arrhenius plot is very old and many phenomenological equations have been published, e.g., Equations (4)–(7), (9) and (10). Some of them have been published and used only a few times but, e.g., the supplement of *C*/(*RT*)^2^ as an addend in Equation (5) (or exp[*C*/(*RT*)^2^] as a multiplicand before logarithmization) causing curvature of the Arrhenius plot) has been used very often as a so-called Bässler-like factor [3,34]. Unfortunately, parameter *C* has no physical meaning and after adding the Bässler factor in Equation (5), parameter *B* loses its previous physical meaning: *B* = −*E* (activation energy).

According to the Arrhenius Equation (1), the reaction rates at very low temperatures are very small but non-zero. This is acceptable for chemical reactions of simple chemicals. On the other hand, many processes whose temperature dependence is also described by the Arrhenius equation, can proceed only above a certain temperature but they cannot proceed below it. In materials science, polymorphous materials can exist in a particular form or crystal structure only above certain temperatures (often within a certain temperature range), e.g., austenite (in equilibrium state) can exist only above the temperature *Ac*_1_. Additionally, plant and animal life can exist above certain individual temperatures. The modified Arrhenius Equation (23) respects these facts: *k* > 0 only for *T* > *T*_0_, for *T* < *T*_0_ is *k* = 0.

**Figure 15:**
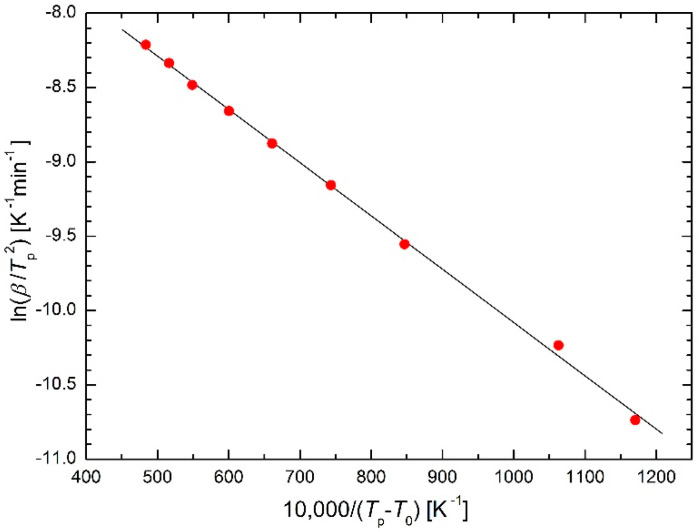
Kissinger plot for Ni-Mn-In-Mg shape memory alloy [5] using the modified Arrhenius Equation (23) as regression function. Replacing 1/*T* axis by 1/(*T* − *T*_0_), a linear plot is obtained instead of a curved plot.

The validity of the modified Arrhenius Equation (23) can be illustrated by drawing in the plot ln*k* vs. 1/(*T* − *T*_0_) instead of in the usual plot ln*k* vs. 1/*T* where the linear course is obtained with the slope −*E*_m_/*R*. This is shown in Figure 4 redrawn in the plot ln*k* vs. 1/(*T* − *T*_0_), see Figure 15. A similar procedure is used in the Weibull plot when a three-parameter Weibull distribution is considered: ln *x*-axis is replaced by ln(*x* − −*x*_0_) axis, see Equation (20).

One of the criteria for the suitability of the regression function is the sum of the squares of the deviations between the measured and calculated values of the observed quantity. These sums are compared in Table 5 for the Kissinger plot for Ni-Mn-In-Mg shape memory alloy [5], see Figures 1 to 4. It can be seen that the worst results are obtained for the twice (“per partes”) used linear dependence and phenomenological function analogous to Equation (5). The lowest sum is obtained for Equation (7) whose derivation is rather problematic, see *Introduction* chapter. Comparable values of the sum are obtained for the two newly presented functions described by Equation (16) (supplemented by Equation (17)) and Equation (23).

Although the symbol *E* denotes the (activation) energy in the original Arrhenius Equation (1), as well as in the modified Arrhenius Equation (23), the two energies differ significantly. Let the energy in Equation (1) henceforth be denoted as *E*_A_ and in Equation (23) as *E*_m_. The activation energy *E*_A_ is usually defined as:

(30) $$E_{A}=-R\frac{\partial \ln k}{\partial (1/T)}=RT^{2}\frac{\partial \ln k}{\partial \;T}$$

therefore, the slope of the Arrhenius plot is equal to −*E*_A_/*R*, see also Equation (2). Differentiation (30) of the modified Arrhenius Equation (23) gives:

(31) $$E_{A}=E_{m}{\left(\frac{T}{T-T_{0}}\right)}^{2}$$

Two limits can be easily determined:

(32) $$\underset{T\to \infty }{\lim}E_{A}=E_{m},\;\underset{T\to T_{0}}{\lim}E_{A}\to \infty$$

as well as three equalities:

(33) $$\begin{array}{ccc} E_{A}=4\;E_{m} & \mathrm{for} & T=2\;T_{0}\\ E_{A}=9\;E_{m} & \mathrm{for} & T=1.5\;T_{0}\\ E_{A}=121\;E_{m} & \mathrm{for} & T=1.1\;T_{0} \end{array}$$

Equation (32) shows that the slope of the modified Arrhenius plot for higher temperatures is approximately equal to the slope of the *classical* Arrhenius plot but approaching *T*_0_ it grows above all limits. Equation (33) implies that *E*_m_ can be orders of magnitude lower than *E*_A_. On the other hand, *E*_m_ is really constant (independent of temperature), while *E*_A_ is dependent on temperature in the curved Arrhenius plot (as invariable constant is only for the classical Arrhenius Equation (1)). For example, for kinetic Equation (4) the differentiation (30) gives the value:

(34) $$E_{A}=R(-B+CT+DT^{2}+2ET^{3}+\ldots )$$

and for Equation (5):

(35) $$E_{A}=-B-2\frac{C}{RT}$$

It is the constant (activation) energy *E*_m_ and the single parameter *T*_0_ (the lowest temperature at which the process under study still takes place), which controls the curvature of the curved Arrhenius plot, which are the main advantages of the newly presented modified Arrhenius Equation (23) compared to a number of other more or less phenomenological relations containing one or often more parameters without any physical meaning.

After *deriving* and verifying the suitability of the modified Arrhenius Equation (23), the author of this paper tried to find some similar equations, i.e., equations containing the exp[*B*/(*T* − *T*_0_)] term or the B/(*T* − *T*_0_) term after logarithmization. They have been found in fields of science far from the fields mentioned above:

- The Vogel−Fulcher−Tammann equation (VFT equation) [35,36,37] is used to describe the viscosity of liquids as a function of temperature, especially near the glass transition:
  (36) $$\eta =\eta_{0}\exp\left(\frac{B}{T-T_{\mathrm{VF}}}\right)$$
  where *η*_0_, *B*, and *T*_VF_ (typically lying about 50 °C below the glass transition temperature) are empirical material-dependent parameters. García-Colín et al. [38] have not very convincingly attempted to build this purely empirical equation on a theoretical basis.
- In the study of shear creep and recovery of elastomers, Conant et al. [39] published an empirical equation for temperature dependence of creep or recovery time *t*_c_:
  (37) $$\ln t_{c}=\frac{C_{\alpha }}{T-b}+C_{\beta }$$
  where *C_α_*, *C_β_*, and *b* are empirical constants. They mentioned the similarity of Equation (36) to the Arrhenius equation and called constant *b* as “correction” term.
- When studying dielectric relaxation in some simple alcohols, Davidson and Cole [40] found that the temperature dependence of relaxation times deviates systematically from the Arrhenius law. Most of their data could be fitted to the expression:
  (38) $$\tau_{0}=A\exp\left(\frac{B}{T-T_{\infty }}\right)$$
  For glycerol and *n*-propanol, these authors even found that the temperature values *T*_∞_ determined from electric relaxation times agree with the temperature values *T*_VF_ (see VFT equation) found in the viscosity studies performed by Tammann and Hesse [37]. This agreement *T*_∞_ = *T*_VF_ means that there are no different specific temperatures for the individual physical quantities, but only one common temperature characterizing the material under study as such.

This means that for the three reciprocal values 1/*η*, 1/*t*_c_ and 1/*τ*_c_, the modified Arrhenius Equation (23) holds. A broader presentation of the interconnection of the Kissinger method used in the study of kinetic processes in materials with many phenomena in other fields of science was made by Vyazovkin in his excellent review [41].

The authors of the Williams-Lander-Ferry model [26] came out of the VFT equation [35,37]. The opposite path is very simple to show. If the reference temperature is identified with glass transition temperature *T*_g_, the VFT equation can be written in the form:

(39) $$\ln\left(\frac{\eta }{\rho T}/\frac{\eta_{g}}{\rho_{g}T_{g}}\right)=-\frac{c_{1}(T-T_{g})}{c_{2}+(T-T_{g})}$$

where *η* is viscosity and *ρ* is the density of the studied liquid. Since the product *ρT* is not very temperature sensitive (especially not compared to the right side of the equation), the simplified left side of the equation ln(*η*/*η*_g_) can be considered. Then Equation (38) can be rewritten into the form:

(40) $$\ln\eta =\ln\eta_{g}-c_{1}+\frac{c_{1}c_{2}}{T-(T_{g}-c_{2})}$$

After the substitutions:

(41) $$\begin{array}{ccc} \ln\eta_{g}-c_{1}=\ln\eta_{0}, & c_{1}c_{2}=B, & T_{g}-c_{2}=T_{\mathrm{VF}} \end{array}$$

logarithmized VFT Equation (36) is obtained.

Soesanto and Williams [42] prefer the WLF equation to the VFT equation (and all other equations of this type, i.e., (36), (37), including the modified Arrhenius Equation (23)). They argue for clarified meaning of the parameters of the WLF equation *T*_g_, *η*_g_, *c*_1_, and *c*_2_. The meaning of the parameters *T*_g_ and *η*_g_ is unquestionable, but parameters *c*_1_ and *c*_2_ are only phenomenological parameters without any physical meaning. In contrast, the parameters in Equation (23) have in principle a clear physical meaning: the meaning of *k*_∞_ is the same as in the original Arrhenius equation, *E* represents some analogy of activation energy (independent of temperature) and *T*_0_ is the temperature characterizing the material under study, common to more physical properties of this material. Moreover, the parameters *k*_∞_, *E*, and *T*_0_ are independent of each other, while the parameters of the WLF equation *T*_g_, *η*_g_, *c*_1_, and *c*_2_ are not (cannot be) independent. This means that for regression, one parameter (usually *T*_g_) must be predetermined and only the remaining three parameters can be calculated in the regression procedure.

As shown above, the regression function (16) accompanied by Equation (17), as well as the modified Arrhenius Equation (23) allow a successful regression of concave Arrhenius plots. On the other hand, their abilities to describe complex chemical reactions are limited. This limitation is quite fundamental and consists of a small number of regression parameters − in principle, it is not possible to accurately describe multi-step reactions using only three or four parameters. Probably the simplest published reaction (11) used in this case had to be further simplified to the reaction with the scheme (14), in fact with the scheme:

(42) $$A\overset{k_{1}\;\mathrm{or}\;k_{2}}{\to }C$$

in order to limit the number of regression parameters in the regression function (16) accompanied by Equation (17) to four. The modified Arrhenius Equation (23) contains only three regression parameters and also cannot be used successfully in the case of more complex phenomena, such as relaxation processes in gas-containing plasmas [43,44]. Overleaf, the dielectric relaxation in some simple alcohols studied by Davidson and Cole [40] has been successfully described using Equation (38), which is actually another form of the modified Arrhenius Equation (23).

## 5. Conclusions

The presented findings can be summarized as follows:

1. In the case where the experimental Arrhenius plot consists of two approximately linear parts, the fit using regression function (16) accompanied by Equation (17) gives a successful result. The use of the regression function (16) is much more suitable than using two *independent* straight lines.
2. For the generally curved Arrhenius plot, the regression function (23) is very suitable. Only one parameter, *T*_0_ with clear physical meaning (the lowest temperature at which the studied process is still running) controls the curvature of the Arrhenius plot. For *T*_0_→0 the modified Arrhenius Equation (23) transitions smoothly into the original Arrhenius Equation (1).
3. The two models presented for a concave-curved Arrhenius plot (super-Arrhenius behavior) can be simply modified for the convex-curved Arrhenius plot (sub-Arrhenius behavior).
4. In addition to chemistry, the newly proposed model (23) can also be used in materials science, food storage, botany, microbiology and even in zoology, as has been demonstrated.
5. In the past, phenomenological equations corresponding to the modified Arrhenius Equation (23) have been used for the temperature dependence description of liquid viscosity, creep or recovery time and dielectric relaxation time. Its validity seems to be very broad in many different fields of science and technology. However, it is necessary to try to interpret the meaning of temperature *T*_0_, as it has been identified with the temperature *Ac*_1_ in the austenitization of SA 508 Gr.3 steel.
6. Also, the WLF equation is essentially equivalent to the modified Arrhenius Equation (23). However, its parameters *T*_g_, *η*_g_, *c*_1_, and *c*_2_ are not independent and for regression one parameter (usually *T*_g_) must be specified in advance and only the remaining three parameters can be calculated in regression procedure.
7. When the temperature dependence of several properties of a single material is studied using the modified Arrhenius Equation (23), the obtained temperature values *T*_0_ represent the only one common temperature characterizing this material as a whole, not its individual properties.

## Figures and Tables

**Table 1:** *T*_0_ temperatures for different conversions corresponding to the plots in Figure 5.

Conversion *α*	0.1	0.35	0.5	0.7	Mean ± s.d.
temperature *T*_0_ [K]	801	943	959	753	864 ± 51
temperature *T*_0_ [°C]	528	670	686	480	591 ± 51

**Table 2:** *T*_0_ temperatures for different conversions corresponding to the plots in Figure 6 (reduced data).

Conversion *α*	0.1	0.35	0.5	0.7	Mean ± s.d.
temperature *T*_0_ [K]	904	997	1005	938	971 ± 17
temperature *T*_0_ [°C]	671	724	732	665	698 ± 17

**Table 3:** Chemical composition of SA508 Gr.3 steel [2].

Element	C	Mn	Si	Ni	Cr	Mo	V	Al	N	Fe
composition wt.%	0.20	1.47	0.17	0.89	0.13	0.51	0.001	0.039	0.014	bal.

**Table 5:** Comparison of sums of squares in the Kissinger plot for Ni-Mn-In-Mg shape memory alloy [5] using the presented equations as regression functions.

Equation(s)	Figure	S
(1) (twice)	1	0.026706
analogy of (5)	1	0.031670
(7)	2	0.003730
(16) and (17)	3	0.009765
(23)	4	0.008962

## Data Availability

Data sharing is not applicable to this article.

## Sample Availability

Samples of the compounds are not available from the author.
